# Genetic variants rs2910164, rs4636297 and rs895819 may contribute to the onset of acute myocardial infarction in Pakistani population

**DOI:** 10.1371/journal.pone.0296025

**Published:** 2024-01-02

**Authors:** Sajjad Ali, Taqweem Ul Haq, Manzar Hussain, Muhammad Uzair, Yasir Ali, Yangchao Chen, Fazal Jalil, Aftab Ali Shah

**Affiliations:** 1 Department of Biotechnology, University of Malakand, Chakdara, Pakistan; 2 Department of Biotechnology, Abdul Wali Khan University Mardan, Mardan, Pakistan; 3 School of Biomedical Sciences, Chinese University of Hong Kong, Hong Kong, China; Suez Canal University, Faculty of Medicine, EGYPT

## Abstract

The most serious type of coronary artery disease (CAD), acute myocardial infarction (AMI), is a major global cause of death. The development of AMI is accompanied by several risk factors. AMI may be caused by variations in the microRNA (miRNA) genes, which have a negative impact on miRNA-mediated regulation of gene expression. The target mRNAs are dysregulated because of these genetic changes in the miRNA genes, which interfere with the vital biological processes that result in AMI. Using allele-specific PCR, the aim of the study is to examine the association of the variants (rs2910164, rs4636297, and rs895819) in *MIR146A*, *MIR126*, and *MIR27A* with AMI susceptibility. A difference in genotype distribution among the patients and control for variation rs2910164 was identified by co-dominant [χ2 = 68.34,2; P value<0.0001], dominant (G/G vs G/C + C/C) [OR = 4.167 (2.860–6.049); P value<0.0001], recessive (C/C vs G/C + G/G) [OR = 0.2584 (0.1798–0.3731); P value<0.0001], and additive models [OR = 3.847 (2.985–4.959); P value<0.0001]. Whereas the association of rs4636297 was investigated by co-dominant [χ2 = 6.882,2; P value = 0.0320], dominant (G/G vs G/A + A/A) [OR = 0.6914 (0.4849–0.9948); P value = 0.0489], recessive (A/A vs A/G + G/G) [OR = 2.434 (0.9849–5.616830); P value = 0.0595], and additive models [OR = 0.7716 (0.6000–0.9918); P value = 0.0433]. Similarly, association of rs895819 was determined by co-dominant [χ2 = 5.277, 2; P value = 0.0715], dominant (G/G vs G/A + A/A) [OR = 1.654(0.9819–2.801); P value = 0.06440], recessive (A/A vs A/G + G/G) [OR = 0.7227 (0.5132–1.022); P value = 0.0748], and additive models [OR = 1.3337 (1.041–1.719); P value = 0.0233]. The results of this study found a significant association of rs2910164 and rs4636297 with AMI and are considered as the risk factor for AMI in the Pakistani population. We observed no significant association of the variant *MIR27A* (rs895819) with AMI incidence.

## Introduction

The exponential increase in the incidence of cardiovascular disease (CVD) has created substantial challenges for healthcare systems globally. CVD is the leading cause of death in the developed and the developing world and a significant contributor to a low quality of life [1]. Incidence of CVDs has increased from 271 million to 523 million in the last 20 years (1999–2019), resulting in 18.6 million deaths globally, accounting for at least 31% of all deaths, with 80% occurring in low- and middle-income nations [2, 3]. In 2020, approximately 19.1 million deaths were attributed to CVD globally and the highest mortality rates attributable to CVD in 2020 were in Eastern Europe and Central Asia, with higher levels also seen in South and Southeast Asia [4]. The South Asian ethnic background has been recognized as a potential predisposing factor for the early onset of cardiovascular diseases [5]. AMI is a common cardiovascular condition that not only negatively impacts patients’ quality of life but also significantly burdens society and families and is the main cause of mortality worldwide for people with CVD [6]. Despite advancements in revascularization techniques and pharmacology, the in-hospital and 5-year mortality rates of AMI remain at 5% and 20%, respectively [7]. The mortality rate for Ishemic Heart Disease (IHD) alone has reached levels ranging from 152 to 204 per 100,000 individuals within the population [8]. The prevalence of this condition is comparatively lower in industrialized nations. At the same time, it tends to rise in developing countries such as South Asia, Latin America (LATAM), and Eastern Europe [9]. People of South Asian origin have a 3–5 times higher risk of morbidity and mortality from heart disease than individuals from other nations. This includes higher rates of AMI at younger ages, primarily due to the influence of risk factors in early life [10].

In Pakistan, Coronary Artery Disease (CAD), including AMI, has emerged as a significant health concern, contributing to 20.28% of all deaths. This alarming statistic has positioned Pakistan at the 18th number globally regarding mortality rates. The age-adjusted death rate stands at 237.98 per 100,000 individuals, highlighting the urgent need for attention and action in combating this widespread issue [11]. Early diagnosis, precise prognostic prediction, and prompt and effective treatment are crucial to enhance patient outcomes, given its rising frequency in recent years, especially among the young population [12]. Clinically, cardiac troponin and creatine kinase-MB (CK-MB) are currently the most often utilized indicators for diagnosing myocardial infarction. Revascularization therapy may assist patients with these biomarkers [13]. However, these indicators are not completely specific for an acute coronary event; while other non-cardiac conditions such as sepsis, chronic kidney injury, and pulmonary embolism are also linked with troponin increase [14]. In recent years, the role of miRNAs in numerous pathophysiological processes in various domains, including cardiovascular disorders, has come into focus as a promising approach [15].

MiRNAs are a group of small nonprotein-coding and single-stranded RNA molecules (19–25 nucleotides) that post-transcriptionally regulate the target mRNAs via miRNA: mRNA pairing [16, 17], following in either target degradation or repression [18], dysregulation of corresponding genes expression [19]. They can regulate more than 30% of protein-coding genes [20, 21]. Molecular components and communication between cells are performed by miRNAs, which play a pivotal role in both normal and pathological conditions [22].

Single Nucleotide polymorphism (SNP), which occurs roughly every 1000–2000 bases, is the most prevalent kind of genetic change. The SNPs in miRNAs can alter miRNA maturation, and expression, and further affect the base-pairing between miRNAs and their target mRNAs [23, 24]. The SNP rs2910164 polymorphism is situated in the precursor stem region of the miR-146a gene, which spans the positive strand of human chromosome 5 and spans nucleotides 160485352 to 160485450 [25]. In its stem structure, this SNP results in a base pair alteration from the G: U pair to the C: U mismatch [26]. Functional studies have demonstrated that the rs2910164 mismatch decreased the expression of mature miRNA, which contributes to AMI vulnerability [27].

The rs4636297 (A>G) SNP is situated in the flanking region of the *MIR126* gene within an intronic part of the epidermal growth factor-like protein 7 (EGFL7) gene and has been connected to post-transcriptional alterations in circulating miR-126 levels [28]. The miR-126 is an endothelial-specific miRNA and is reported as linked to endothelial activation and damage [29]. Recent research indicated a significant relationship (p < 0.05) between the rs4636297 and rs1140713 SNPs of the miR-126 gene and various clinical indicators in patients diagnosed with AMI [30]. On the negative strand of chromosome 19, another SNP, rs895819, can be found in the mature(hsa-miR-27a) of the *MIR27A* gene from nucleotides 13836440 to 13836517 [31]. A frequent polymorphism (rs895819) was identified in the coding region of *MIR27A* gene. Previous studies have confirmed a strong correlation between rs895819 and the risk of MI [32]. Therefore, the current study investigated the potential role of rs2910164, rs4636297, and rs895819 polymorphisms as a susceptibility factor for acute myocardial infarction in Pakistani population. The SNP in the studied miRNA genes polymorphism was selected using the International HapMap Project (http://www.hapmap.org), dbSNP (http://www.ncbi.nlm.nih.gov/projects/SNP/) and Mirbase (http://microrna.sanger.ac.uk).

## Materials and methods

### Ethical standards, inclusion and exclusion criteria, and sample collection

The current study followed the Helsinki Declaration. Verbal informed consent was taken from all healthy individuals and patients. The current study was approved by the Advanced Studies and Research Board (ASRB), University of Malakand in its 59^th^ meeting held on 17^th^ December, 2020. This study included 400 patients diagnosed with AMI in accordance with the Third Universal Definition of MI [33]. The patients included in this study fulfilled the following specified criteria. Cardiac markers, specifically troponin, clinically detected when their levels exceed the 99^th^ percentile value of the upper limit of the normal reference value; symptoms of myocardial ischemia followed by coronary artery blood supply; Prolonged chest pain lasting more than 30 minutes, characterized by a feeling of tightness, radiate to various regions such as the extremities, neck, jaw, and back; the appearance of pathological Q-waves, new substantial ST-segment up or down or T-wave changes on the ECG, and imaging evaluation revealing recent myocardial loss or new segmental wall motion abnormalities. In this current study, patients with a medical history of non-coronary cardiac disorders, poorly controlled blood pressure, chest pain caused by trauma or drug use, prior coronary bypass surgery, and other contraindications, such as cancer, liver or renal failure were not included. The control group was randomly selected from individuals without a history of cardiovascular events and with normal physical examination results for cardiovascular system function. The demographic information, such as age, gender, and medical history, including blood pressure, and type of MI, were recorded upon admission, as shown in Table 1. In ethylenediaminetetraacetic acid (EDTA) tubes, 5CC whole blood was drawn from AMI patients and healthy controls. The phenol/chloroform procedure was used to extract genomic DNA from the extracted blood samples [34]. The genomic DNA was dissolved in 50ul of nuclease-free water and stored at 4° Celsius.

**Table 1:** Baseline characteristics of MI patients and healthy controls including blood pressure.

Categories	Mean Age (Year)	Gender	Mean B.P Systolic/Diastolic (mmHg)	Type of MI (Trop I > 0.03 ng/Ml)
**Case (AMI)**	± 57 (25–90)	Male = 262 Female = 138	± 145/95 (100/70-180/110)	NSTEMI = 222 STEMI = 178
**Controls**	± 50 (27–85)	Male = 113 Female = 87	± 118/80 (110/85-130/90)	Nill

BP: Blood pressure, MI: Myocardial Infarction

### Genotyping of Selected SNPs using T-ARMS PCR Assay

The miRbase (http://www.mirbase.org) contains details about the genetic context of 1917 precursors and 2654 mature human miRNAs. From miRbase, pertinent information about the SNPs included in the present study was acquired [35].

Single nucleotide polymorphisms (SNPs) can be genotyped using the tetra-primer amplification refractory mutation system-polymerase chain (T-ARMS-PCR) procedure. The tetra-primer ARMS-PCR uses four primers in one PCR to verify the genotype (as shown in Table 2). At the start of the reaction, two outside primers that are not allele-specific amplify the region containing the SNP. The two allele-specific primers (inner primers) generate allele-specific fragments, which use the outer primers as a template. They lessen the likelihood of false-positive results in this way [36, 37].

**Table 2:** List of T-ARMS primers used for genotyping rs2910164, rs4636297 and rs895819.

SNP Name	Primer Sequences	PCR Product
rs2910164	FO-GGCCTGGTCTCCTCCAGATGTTTAT	364bp
	RO-ATACCTTCAGAGCCTGAGACTCTGCC	
	Fl-ATGGGTTGTGTCAGTGTCAGACGTC	169bp
	RI-GATATCCCAGCTGAAGAACTGAATTTGAC	249bp
rs4636297	FO-GGATAGGTGGGTTCCCGAGAACTG	327bp
	RO-TCTCAGGGCTATGCCGCCTAAGT	
	FI-TTCAAACTCGTACCGTGAGTAATAATGAGC	156bp
	RI-GTTTTCGATGCGGTGCCGTGGAAGA	225bp
rs895819	FO-AGGGGAGGTGTCCCCAAATCTCATTACC	479bp
	RO-CCTGTTCCTGCTGAACTGAGCCAGTGTA	
	FI-CTGCTTGTGAGCAGGGTCCCCA	213bp
	RI GGAACTTAGCCACTGTGAACACGACTTTGC	318bp

Using the online tools provided by Primer 1 (http://primer1.soton.ac.uk/primer1.html), primers for the specified SNPs were designed [38]. For each SNP, two inner allele-specific primers (FI and RI) and two outer primers (FO and RO) were used for genotyping the rs2910164, rs4636297, and rs895819 polymorphisms.

Fig 2 shows a diagrammatic representation of the stem-loop structure containing the SNP under investigation, rs2910164. Table 3 provides information about names, stem loop sequence, mature miRNA sequences, chromosomal location, MAF, and effects of the studied SNPs.

**Table 3:** Shows the list of studied SNPs rs2910164, rs4636297, and rs895819, their official name, stem-loop sequence, maturmiRNA sequences, chromosomal location, MAF, and their consequences.

salSNP ID	miRNA Gene Name	Stem = loop sequnce	mature miRNA	Genomic context	Coded Allele	Other allele	MAF	Consequences
rs2910164	*MIR146A*	CCGAUGUGUAUCCUCAGCUUUGAGAACUGAAUUCCAUGGGUUGUGUCAGUGUCAGACCUCUGAAAUUCAGUUCUUCAGCUGGGAUAUCUCUGUCAUCGU	hsa-miR-146a-3p CCUCUGAAAUUCAGUUCUUCAG	Chr5:160485352-160485450 [+]	C	G	0.40	Mature miRNA variation
			hsa-miR-146a-5p UGAGAACUGAAUUCCAUGGGUU					
rs4636297	*MIR126*	CGCUGGCGACGGGACAUUAUUACUUUUGGUACGCGCUGUGACACUUCAAACUCGUACCGUGAGUAAUAAUGCGCCGUCCACGGCA	hsa-miR-126-3p UCGUACCGUGAGUAAUAAUGCG	Chr9:136,670,602-136,670,686 [+]	A	G	0.45	Intron Variant
			hsa-miR-126-5p CAUUAUUACUUUUGGUACGCG					
rs895819	*MIR27A*	CUGAGGAGCAGGGCUUAGCUGCUUGUGAGCAGGGUCCACACCAAGUCGUGUUCACAGUGGCUAAGUUCCGCCCCCCAG	hsa-miR-27a-3p UUCACAGUGGCUAAGUUCCGC	chr19: 13836440–13836517 [–]	T	A, C, G	0.49	Non-coding transcript exon variant
			hsa-miR-27a-5p AGGGCUUAGCUGCUUGUGAGCA					

### T-ARMS PCR condition for the genotyping of rs2910164, rs4636297 and rs895819

A 25μL solution was made using each primer to prepare for the T-ARMS-PCR reaction. The solution was composed of 13μL of master mix (DreamTaq Green PCR Master Mix (2X), Catalog No. K1081, Thermo Scientific USA) for each solution; primer concentration in each solution was 6μL for 146a (rs2910164), 5μL for 126 (rs4636297), and 4μL for 27a (rs895819), respectively; with 2μL of DNA (1ng/μL) from the sample in each solution. The remaining volume was filled with nuclease-free ddH2O. The initial step involved denaturation at 95 °C for 5 minutes for genotying of *MIR146A* (rs2910164), and 10 minutes each for *MIR126* (rs4636297), and *MIR27A* (rs895819). Subsequently, 35 cycles of denaturation at 95°C for 30 seconds, 40 cycles of 35 seconds, and 30 cycles of 40 seconds were completed for each primer, respectively. The annealing temperature was set at 63°C for 30 seconds for rs2910164, 58°C for 40 seconds for rs4636297, and 63°C for 30 seconds for rs895819. The extension step was performed at 72°C for 25, 43, and 40 seconds, respectively. The final extension was completed at 72°C for 10 minutes for each primer. A 2% agarose gel was used to run the PCR products, and a UV-Trans illuminator (Wealtec Bioscience, Meadowvale Way, Sparks NV, USA) was used to visualize the PCR product.

### Sanger sequencing

The results of T-ARMS PCR were also confirmed by the Sanger sequencing. For Sanger sequencing, DNA samples were amplified using conventional PCR techniques. Nanodrop quantification of PCR product was performed by Multiskan microplate reader Catalog No: N12391M2, ThermoFisher Scientific, USA) to assess purity while quality was assessed by agarose gel electrophoresis. Purification of the PCR product was done by ExoSap IT (Catalog No: 75001.4X.1.ML, Thermofisher Scientific USA). The cycle sequencing reaction was performed on SimpliAmp thermal cycler (Catalog No: A24811, Thermofisher Scientific USA). Big Dye X Terminator purified the cycle sequencing product, and the purified product was sequenced on the SeqStudio genetic analyzer (Catalog No: A35644, Thermofisher Scientific USA). The Accelrys Gene version 2.5 program was used to analyze the chromatograph and to find out the sequence variation. The PCR primers used for miR-146a (rs2910164) sequencing were 5’-ATGAGTGCCAGGACTAGACC-3’ and 5’- CTCTCCAGGTCCTCAAGCC-3’.

### Statistical analysis

Hardy–Weinberg Equilibrium (HWE) was used in the present study. Chi-square analysis and Fisher exact test were employed to determine the allelic and genotypic frequencies of rs2910164, rs4636297, and rs895819 variants in *MIR146A*, *MIR126*, and *MIR27A*, respectively, among AMI patients and healthy individuals. The Odds ratios with a 95% confidence interval were used to ascertain the relationship between the SNPs and AMI. The inheritance models (Co-dominant, dominant, recessive and additive) were used to assess the risk of AMI. A P-value of 0.05 was used to establish the statistical level of significance. GraphPad Prism 8.0 was used for data analysis.

## Results

### Association of rs2910164 with the risk of acute myocardial infarction

The genotypic frequencies for the variants were calculated using the Hardy-Weinberg Equilibrium. To determine the genotyping error rate, the subject study (10%) was re-genotyped. To statistically corroborate the polymorphisms, four distinct inheritance models co-dominant, dominant, recessive, and additive were used. For variation rs2910164, co-dominant [χ2 = 68.34, 2; P value<0.0001], dominant (G/G vs G/C + C/C) [OR = 4.167(2.860–6.049); P value<0.0001], recessive (C/C vs G/C + G/G) [OR = 0.2584 (0.1798–0.3731); P value<0.0001], and additive models [OR = 3.847 (2.985–4.957); P value<0.0001]. The genotype frequency distribution revealed that the genotype GG in cases and control were found at 61.25% (n = 400) and 27.5% (n = 200), respectively. The CC genotype in cases was 23.5% (n = 400) and 55% (n = 200) in the control while the genotype GC was found 14.75% in cases (n = 400) and 17.5% in the control group (n = 200) as shown in Table 4.

**Table 4:** Inheritance models for investigating the association of rs895819, rs4636297 and rs2168518 with AMI.

Gene (Accession Number)	Statistical Models	Genotypes	Cases	Control	Odds Ratiο (95% Cl)	χ2-value, df	p-value
***MIR146A*** **(rs2910164)**	Co-dominant	GG GC CC	245(61.25%) 59(14.75%) 96(24%)	55(27.5%) 35(17.5%) 110(55%)	---	68.34,2	<0.0001
	Dominant	GG GC+ CC	245(61.25%) 155(38.75%)	55(27.5%) 145(72.5%)	4.167 (2.860 to 6.049 )	---	<0.0001
	Recessive	CC GC + GG	96(24%) 304(76%)	110(55%) 90(45%)	0.2584 (0.1798 to 0.3731 )	---	<0.0001
	Additive	G C	549(137.25%) 251(62.75%)	145(72.5%) 255(127.5%)	3.847(2.985-to 4.959)	---	<0.0001
***MIR126*** ***(rs4636297)***	Co-dominant	GG AG AA	112(28%) 260(65%) 28(7%)	72(36%) 122(61%) 6(3%)	---	6.882, 2	0.0320
	Dominant	GG AG+AA	112(28%) 288(72%)	72(36%) 128(64%)	(0.6914) (0.4849-to 0.9948)	---	0.0489
	Recessive	AA GG+AG	28(7%) 372(93%)	6 (3%) 194(97%)	2.434 (0.9849–5.616)	---	0.0595
	Additive	GA	484(121%) 316(79%)	266(133%) 134(67%)	0.7716 (0.6000to 0.9918)		0.0433
***MIR27A*** **(rs895819)**	Co-dominant	GG GA AA	65(16.25%) 192(48%) 143(35.75%)	21(10.5%) 92(46%) 87(43.5%)		5.277,2	0.0715
	Dominant	GG GA+ AA	65(16.25%) 335(83.75%)	21(10.5%) 179(89.5%)	1.654 (0.9819to-2.801)	---	0.0640
	Recessive	AA GA + GG	143(35.75%) 257(64.25%)	87(43.5%) 113(56.5%)	0.7227 (0.5132-to 1.022)	---	0.0748
	Additive	GA	322(80.5%) 478(119.5%)	134(67%) 266(133%)	1.337 (1.041-to 1.719)	---	0.0233

**Fig 1:**
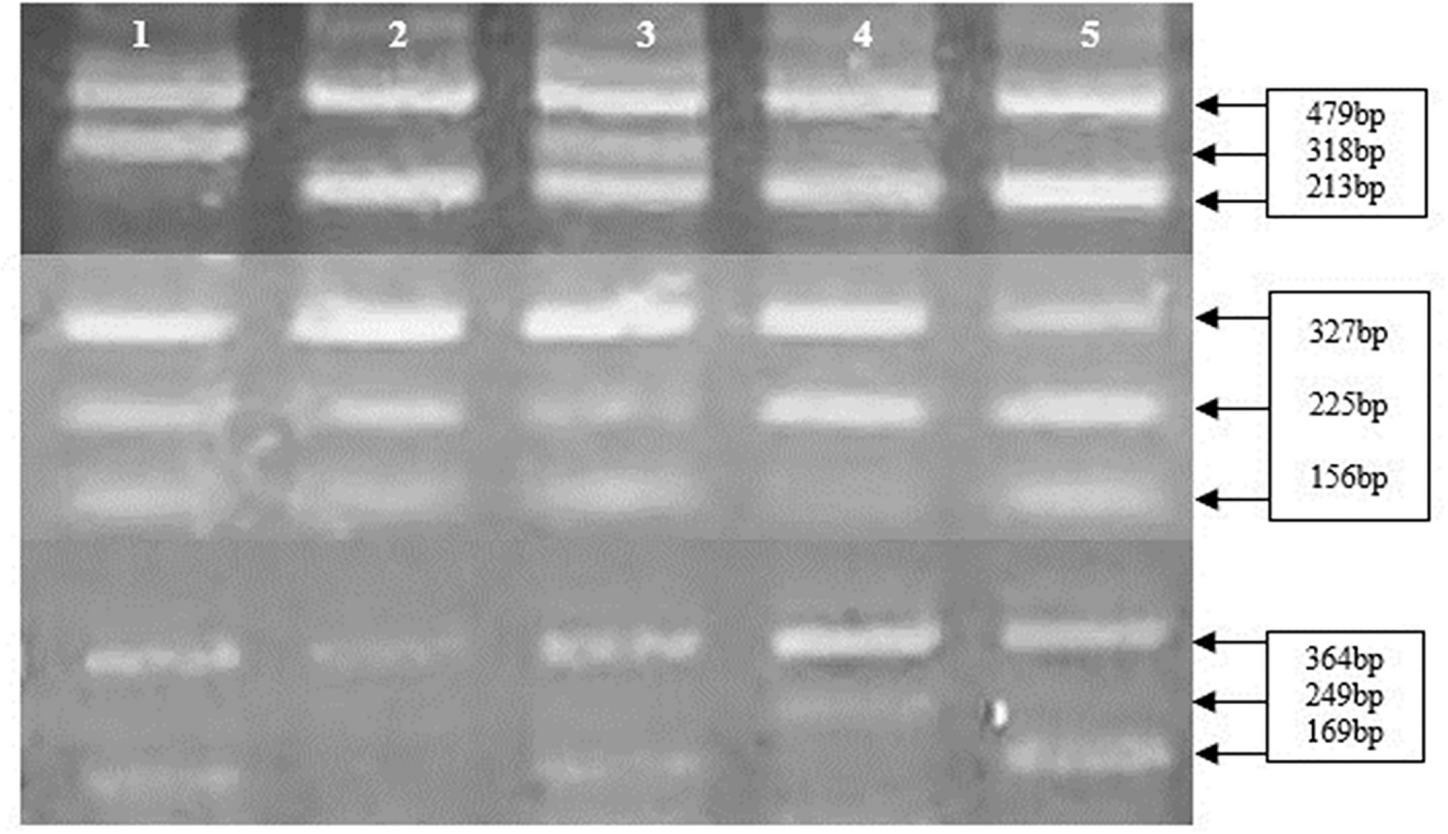
Showed T-ARMS-PCR for the detection of SNPs rs2910164 (upper), rs4636297 (middle), and rs895819 (lower) *MIR146A*, *MIR126*, and *MIR27A* genes.

**Fig 2:**
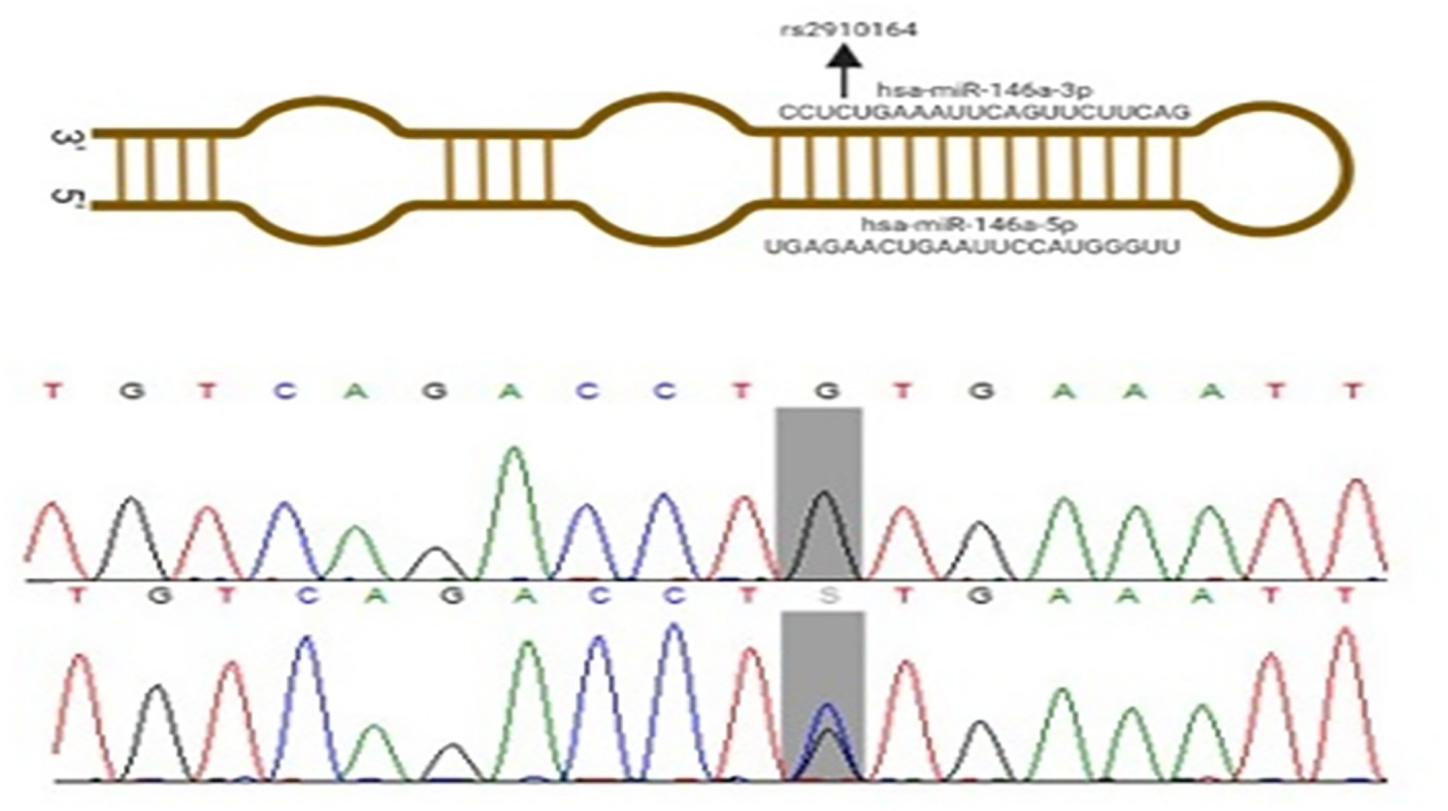
Showed a diagrammatic representation of the stem loop structure of miRNA containing the SNP (rs2910164) (upper). Validation assay to confirm T-ARMS PCR result of rs2910164 (G>C) using Sequencing (lower).

The statistical analysis of the variant using all four inheritance models showed significant variations in the frequency of this miRNA polymorphism between the patient and control groups, indicating that it should be regarded as a risk factor for AMI in the studied population. Fig 1 shows T-ARMS-PCR for the detection of SNPs rs2910164 (upper), rs4636297 (middle, and rs895819 (Lower) *MIR146A*, *MIR126*, and *MIR27A* genes. The results of T-ARMS PCR were further validated by Sanger Sequencing (Fig 2).

### Association of rs4636297 with increased risk of acute myocardial infarction

A significant difference was observed for rs4636297 through co-dominant model analysis [χ2 = 6.882, 2; P value = 0.0320], dominant (G/G vs G/A + A/A) [OR = 0.6914 (0.4849–0.9948); P value = 0.0489], and additive models [OR = 0.7716 (0.6000–0.9918); P value = 0.0433] were found significant with the risk of AMI. The genotype frequency distribution revealed that the genotype GG in cases and control were found at 28% (n = 400) and 36% (n = 200), respectively. The AA genotype in cases was 7% (n = 400) and 3% (n = 200) in the control while the genotype AG was found 65% in cases (n = 400) and 61% in the control group (n = 200). The recessive statistical model (A/A vs A/G + G/G) [OR = 2.434 (0.9849–5.616); P value = 0.0595] for the variant rs4636297 was found not significant.

### Association of rs895819 with increased risk of acute myocardial infarction

The genotypic frequency for SNP rs895819 was calculated by co-dominant [χ2 = 5.277, 2; P value = 0.0715], dominant (G/G vs G/A + A/A) [OR = 1.654 (0.9819–2.801); P value = 0.0640], recessive (A/A vs A/G + G/G) [OR = 0.7227(0.5132–1.022); P value = 0.0748], and additive models [OR = 1.337 (1.041–1.719); P value = 0.0233]. The genotype frequency distribution revealed that the genotype GG in cases and control were found at 16.25% (n = 400) and 10.5% (n = 200), respectively. The AA genotype in cases was 35.75% (n = 400) and 43.5% (n = 200) in the control while the genotype GA was found 48% in cases (n = 400) and 46% in the control group (n = 200). The significant difference was only observed in the allele frequency distribution model, while the other three models were found non-significant.

### The impact of variants on the secondary structure of miRNAs

The centroid secondary structure of both the reference and variant precursor miRNAs was formed, and the MFE (minimal free energy) was obtained as given in Table 5. This method allowed us to assess the effect of SNP on the secondary structure’s stability. The current data shows that the variant can generate significant structural changes in the precursor miRNA.

**Table 5:** Information about the free energy of the thermodynamic ensemble, the frequency of the MFE structure in the ensemble, the ensemble diversity, the optimal secondary structure with a minimum free energy and the centroid secondary structure of studied reference SNPs and their mutated variants.

Property	Reference	Mutated	Reference	Mutated
	*MIR146A* rs2910164	*MIR146A* rs2910164	*MIR27A* rs895819	*MIR27A* rs895819
Free energy of the thermodynamic ensemble	-40.49 kcal/mol	-43.44 kcal/mol.	-38.24 kcal/mol	-38.28 kcal/mol.
The frequency of the MFE structure in the ensemble	8.87%	9.72%.	15.62%.	14.84%.
The ensemble diversity	5.40	4.98	4.41	4.55
The optimal secondary structure with a minimum free energy	-39.00 kcal/mol	-42.00 kcal/mol	-34.40 kcal/mol	-34.40 kcal/mol
The centroid secondary structure	-39.00 kcal/mol	-42.00 kcal/mol	-37.10 kcal/mol	-37.10 kcal/mol

## Discussion

Polymorphisms in the human genome cause a variety of phenotypes. Previously it was investigated that SNPs in miRNAs were associated with cardiovascular diseases including AMI [39]. In the current study, SNPs (rs2910164, rs4636297, and rs895819) in*MIR146A*, *MIR126* and *MIR27*A, respectively, were genotyped and their relationships to AMI were examined in Pakistani population. The miR-146a has been documented as a prospective regulator in numerous mechanisms of oxidative stress, metabolism, immunoreaction, inflammation, and several other diseases, including cerebrovascular and cardiovascular conditions [40, 41]. Previously, the clinical significance of rs2910164 in various varieties of cancer has been well established; for instance, papillary thyroid cancer [42], non-small-cell lung cancer [43], head and neck cancer [44], oral carcinoma [45] and breast cancer [46]. The present study revealed that the SNP rs2910164 was a risk factor for the development of AMI. Another study reported that patients with ST-segment elevation myocardial infarction (STEMI) had raised levels of miR-146a compared to controls and had a higher chance of having major adverse cardiovascular events than those with lower levels of the miRNA [47]. Recent research indicates that the level of miR-146a is significantly elevated in the exosomes of STEMI and NSTEMI patients using the RT-qPCR technique and is associated with an inflammatory response [48]. It has been reported that miR-146a levels were shown to be greater in AMI patients of the Chinese population, and the GG phenotype of *MIR146A* rs2910164 was linked with a higher risk of AMI than the CC phenotype [49]. In contrast to our findings, a previous study found that the *MIR146A* rs2910164 variant is associated with a reduced risk of CHD in the Asian population [50]. A recent study by Mehrjardi et al. revealed no statistically significant correlation between the *MIR146A* gene polymorphism (rs2910164) and CAD within the Iranian population [51].

The association of *MIR126* (rs4636297) with AMI was also investigated and the results indicated association with the AMI. Prior research has documented the identification of circulating miR-126 as a promising biomarker for various conditions, including type 2 diabetes [52], non-alcoholic fatty liver disease [53], and lung cancer [54]. Furthermore, it has been reported that it may also reflect the development of cardiovascular disease [55]. The same findings was also reported by Xue et al. (2019), that the level of miR-126-5p is significantly increased in AMI patients within 4 hours of the onset of chest pain in the Chinese population and is considered a biomarker for the early diagnosis of AMI [56]. The recent study reported that elevated level of miR-126-3p have been shown to serve as a reliable predictor of cardiovascular events including MI, while also providing a protective effect on endothelial cells against vascular shocks [30]. Zong et al. performed the receiver operating characteristic (ROC) curve and the area under the ROC curve (AUC) (with a P< .05 and an AUC > 0.6) analysis, to evaluate the diagnostic value of miR-126 to differentiate the AMI and non-AMI control group, and reported the increased level of miR-126 in the AMI patients as compared to the control group [57], Contrary to the above studies, it was reported in the previous work conducted in the Chinese population that there was no significant correlation between polymorphisms in pri-miR-26a-1, pri-miR-100, pri-miR-126, and pri-miR-218 and the risk of developing MI, employed allelic and known genetic models [58]. It has been observed in the population of Poland that levels of miR-126 were lower in patients with myocardial damage during the trans-coronary passage [59]. In cardiac muscle cells, the miR-126 is involved in the process of repair by increasing the angiogenesis and progenitor cell recruitment found at the injury sites and during the trans coronary passage through these damaged vessels, its levels drop in the blood [60]. Several studies have shown that the level of miR-126 decrease in stable coronary artery disease [61] and AMI patients as compared to the healthy subjects [62]. The study of plasma miRNAs in congestive heart failure revealed that miR-126 plasma concentration declined with age and was downregulated by unfavorable conditions of congestive heart failure (CHF) [63]. The same finding was also reported in another study, and it was noted that the plasma level of miR-126 was downregulated in AMI patients after the onset of symptoms of AMI [64]., I.

In addition to being reported as an oncogenic miRNA, the investigated miR-27a (rs895819) has been shown to regulate lipid metabolism by modulating the expression of many genes involved in lipid metabolism [65]. It has been reported that the pre-miR-27a rs895819 polymorphism is significantly linked with the risk of type-2 diabetes (T2DM) [66]. Additionally, miR-27a had a special role in malfunctioning endothelial cells (ECs), which may cause cardiovascular diseases such as atherosclerosis, CAD, and MI [67]. The current study investigated the role of rs895819 in the occurrence of AMI and indicated no association with AMI. According to a previous investigation by Jansen et al., numerous circulating microRNAs, including miRNA-126, miRNA-222, miRNA-let7d, miRNA-21, miRNA-20a, miRNA-27a, miRNA-92a, miRNA-17, miRNA-130, and miRNA-199a were not significantly related to cardiovascular events despite their involvement in vascular activities [61]. In heart disease, MiR-27a is downregulated in coronary sinus blood samples from chronic heart failure patients [68] and can control extracellular matrix changes, inhibit cardiac fibrosis and participate in stem cell development into cardiomyocytes [69, 70]. According to research by Cai et al., the AG and AA haplotype of miR-27a’s rs895819 reduced the possibility of MI in Chinese Han population [58]. In another study, it has been reported that the level of miR-27b between the STEMI and control groups did not vary significantly [71]. The same findings were also reported by Xue et al. (2019), who examined the diagnostic efficiency of the circulating miRNAs, including miR-27A, in AMI and found no substantial difference in miR-27A level between AMI patients and controls [72]. In contrast to these studies, early findings on the expression of many circulating miRNAs after acute myocardial infarction (AMI), the miR-27a levels increased after AMI and are associated with left ventricular contractility [73]. According to a previous study, MIR-27a has also been linked to inhibiting the expression of lipid metabolism genes, leading to lipid metabolism dysfunction, which results in the critical pathogenesis of MI [65].

### Limitation of the study

Nevertheless, a few issues with the current study must be addressed. First, this was a hospital-based case-control study. Participants could have perfectly represented the population as a whole if samples were using cluster sampling approach. Second, the small sample size of our investigation limited its statistical efficacy. Third, Multiple comparison analysis was not done in the current study.

## Conclusion

The MIR146a rs2910164 and MIR126 (rs4636297) polymorphism showed an association with AMI in the studied population, while the) MIR27a (rs895819) is not associated with AMI.
